# KCNT1 (Slack/Slo2.2) and KCNT2 (Slick/Slo2.1) Dysregulation in Intellectual Disability and Behavioral Phenotypes: A Systematic Review

**DOI:** 10.7759/cureus.93770

**Published:** 2025-10-03

**Authors:** Karthika Murugesan, Areeba Kabir, Basel T Tomalieh, Jessica Torres, Alousious Kasagga, Delvy Rebellow

**Affiliations:** 1 Biology, University of North Texas, Denton, USA; 2 Psychiatry and Behavioral Sciences, California Institute of Behavioral Neurosciences and Psychology (CIBNP), Fairfield, USA; 3 Respiratory Medicine, Aintree University Hospital, Liverpool, GBR; 4 Pathology, Universidad Tecnológica Centroamericana (UNITEC), Tegucigalpa, HND; 5 Pathology, Peking University, Beijing, CHN; 6 Internal Medicine, Malankara Orthodox Syrian Church Medical College, Kolenchery, IND; 7 Internal Medicine, California Institute of Behavioral Neurosciences and Psychology (CIBNP), Fairfield, USA

**Keywords:** cognitive impairment, developmental delay, intellectual disability, kcnt1 (slack/slo2.2), kcnt2 (slick/slo2.1)

## Abstract

Potassium channels, particularly the sodium-activated Slo2 (Slack/KCNT1) channels, play a critical role in regulating neuronal excitability and protein synthesis. Emerging evidence suggests a strong association between Slo2 channel dysfunction and the development of intellectual disability (ID), including conditions like Fragile X syndrome and early-onset epileptic encephalopathies. This systematic review was designed and reported in accordance with the Preferred Reporting Items for Systematic Reviews and Meta-Analyses (PRISMA) 2020 guidelines. Studies focusing on molecular, genetic, physiological, or clinical aspects of Slo2 channel function, regulation, or associated disorders were considered. A comprehensive literature search was conducted across multiple electronic databases: PubMed, Europe PubMed Central, Semantic Scholar, Directory of Open Access Journals (DOAJ), ScienceDirect, and Google Scholar. Search terms combined keywords and Medical Subject Headings (MeSH) relevant to Slo2 channels, KCNT1/KCNT2, sodium-activated potassium channels, ID, and neurological disorders. In total, eight studies met the inclusion criteria and were included in the final synthesis. This systematic review synthesizes findings from eight key studies published between 2010 and 2025, encompassing experimental animal models, narrative reviews, and molecular investigations with full-text availability. Each included study underwent methodological quality assessment using validated critical appraisal tools appropriate to its design. Quality assessments were performed using tools such as AMSTAR 2 applied for systematic reviews, cell study-OHAT-inspired for cell experimental studies, and SYRCLE's RoB for animal studies. The review highlights the dual impact of both gain- and loss-of-function KCNT mutations on cognitive function, with gain-of-function mutations often associated with more severe phenotypes. It further explores the molecular mechanisms involving FMRP and CYFIP1 interactions with Slack channels, implicating dysregulated protein translation as a contributor to ID. The literature shows that Slo2 potassium channels are implicated in ID, with both gain- and loss-of-function mutations contributing to cognitive impairment. The molecular mechanisms linking these mutations to clinical phenotypes remain incompletely defined. The findings support a growing consensus that Slo2 channelopathies represent a significant and under-recognized cause of neurodevelopmental impairment, warranting further translational research and therapeutic exploration.

## Introduction and background

Intellectual disability (ID) is a lifelong neurodevelopmental disorder, with onset during childhood or adolescence, characterized by significant impairments in intellectual functioning, typically indicated by an intelligence quotient (IQ) below 70, and adaptive behavior, which encompasses conceptual, social, and practical skills. ID affects approximately 1%-3% of the global population. Although the precise underlying mechanisms have yet to be determined, current research identifies both genetic mutations and environmental exposures as major contributing factors [1]. Among the various genetic causes, several syndromic and non-syndromic forms of ID have been described, with Fragile X syndrome standing out as a key example.

Fragile X syndrome is the most common inherited cause of ID, affecting approximately 1 in 4,000 males and 1 in 8,000 females, and is often associated with cognitive impairment, seizures, and anxiety. Fragile X syndrome results from a CGG short tandem repeat (STR) expansion in the FMR1 gene, resulting in altered gene expression. The disease severity correlates with the length of the CGG tract [2]. The FMR1 gene encodes the fragile X mental retardation protein (FMRP) that is expressed at high levels in neurons [3]. FMRP interacts with a range of mRNA and intracellular proteins. One of the FMRP-interacting proteins is the (KNa) Slo2 channel, which is a Na-activated potassium channel. FMRP interacts with the cytoplasmic C-terminal domain of Slo2 channel and enhances the channel activity. Absence of FMRP causes fragile X syndrome, the most common cause of inherited ID [4-6].

The loss of FMRP, as seen in fragile X syndrome, disrupts the regulation of Slo2 potassium channel activity, thereby linking genetic mutations to impaired neuronal excitability and providing essential insights into the molecular basis of ID. Potassium channels are crucial in regulating neuronal excitability and synaptic transmission. The KCNT gene encodes Slo2 potassium channels, specifically Slo2.1 (also known as Slick) and Slo2.2 or Slack, which are widely distributed and exhibit marked abundance in neurons. Sodium ions activate Slo2 channels, contributing to the delayed outward currents and modulating hyperpolarization, which helps shape neuronal excitability [6]. Slo2 channels regulate physiological processes such as maintaining temporal accuracy in brainstem auditory neurons, neuronal burst regulation in sensory neurons, and overall neuronal development [7]. Slo2 channels engage with downstream cytoplasmic signaling pathways, and disruption of this interaction may underlie the uncommon co-occurrence of ID and epilepsy [8-10].

While Slo2 channels are recognized as essential for maintaining normal cognitive function, their precise contribution to ID is still poorly understood. Most studies have emphasized their role in seizure generation, leaving cognitive aspects underexplored. This review systematically examines the link between Slo2 potassium channels and ID, with a primary focus on cognitive impairments rather than epilepsy. It integrates current understanding of underlying mechanisms, such as gain- or loss-of-function mutations, along with recent advances in animal models, therapeutic developments, and identifies existing gaps in the literature.

## Review

Methodology

The present review was conducted in accordance with the PRISMA 2020 guidelines. The research question was structured using the Population, Intervention, Comparison, and Outcome (PICO) model. Relevant studies published from 2010 to 2025 were retrieved through systematic searches across five databases: PubMed, Google Scholar, Semantic Scholar, Europe PMC, and DOAJ. For each database, tailored search strategies incorporating keywords and Medical Subject Headings (MeSH) were applied to maximize the identification of eligible literature.

Inclusion Criteria

This systematic review considered studies published in English over the last 15 years with full-text availability. Eligible studies included those investigating potassium channels, with a particular focus on KCNT1/Slo2 channels in the context of ID or cognitive impairment. Both human studies and animal models were included, encompassing a range of study designs, including experimental studies, observational studies, systematic reviews, and narrative reviews.

Exclusion Criteria

Studies focused solely on epilepsy without reporting cognitive or intellectual outcomes were excluded, as this review specifically addressed the role of KCNT1/Slo2 channels in cognitive impairment. Publications in languages other than English, as well as editorials, letters, and conference abstracts, were excluded due to the limited methodological detail and outcome data. Articles published more than 15 years ago were excluded to ensure the synthesis reflected current scientific knowledge and advances. Materials without peer-reviewed full-text availability, such as websites, blogs, posters, and presentations, were also excluded because of concerns regarding reliability and data quality. These criteria were applied to minimize bias and maintain rigor in the review process.

Screening and Selection Process

Data extraction and screening were performed independently by two reviewers. All relevant studies were imported, and duplicates were removed using the EndNote reference manager (Clarivate, London, UK). The remaining articles were manually screened by reviewing titles and abstracts. The shortlisted studies were then assessed for full-text availability and adherence to the inclusion criteria. Only articles meeting all criteria were included in the final analysis.

Search Strategy

A literature search was carried out across multiple electronic databases, including PubMed, Europe PMC, Semantic Scholar, DOAJ, ScienceDirect, and Google Scholar. The search combined relevant keywords and MeSH related to Slo2 channels, KCNT1/KCNT2, sodium-activated potassium channels, intellectual disability, and neurological disorders. After applying the inclusion criteria, eight studies were selected for final analysis. This systematic review integrates findings from these eight studies, published between 2010 and 2025, covering experimental animal models, narrative reviews, and molecular investigations, all of which had full-text availability (Table 1).

**Table 1:** Literature search strategy across selected electronic databases This table summarizes the databases searched, key concepts/keywords used, detailed search strategies, applied filters (timeframe: last 15 years), and the number of records retrieved for studies related to Slo2 channels, KCNT1/KCNT2, and associated neurodevelopmental disorders including intellectual disability. PMC: PubMed Central, DOAJ: Directory of Open Access Journals.

Databases	Keywords	Search strategy	Filters	Search result
PubMed	Slo2 channels and intellectual disability, developmental delay	(KCNT1 OR "Slo2" OR "Slack channel" OR "Sodium-activated potassium channel") AND ("intellectual disability" OR "mental retardation" OR "cognitive impairment" OR "developmental delay")	Last 15 years	48
Google Scholar	Slo2 channels and intellectual disability	FMRP AND KCNT1 AND "intellectual disability"	Last 15 years	143
Semantic Scholar	Slo2 channels and intellectual disability, cognitive impairment	"KCNT1 intellectual disability", "Slack channel cognitive impairment", "Potassium channel neurodevelopmental disorder"	Last 15 years	34
Europe PMC	Slo2 channels and intellectual disability	(KCNT1 OR "Slo2" OR "Slack channel" OR "Sodium-activated potassium channel") AND ("intellectual disability" OR "mental retardation" OR "cognitive impairment" OR "developmental delay") NOT epilepsy	Last 15 years	73
DOAJ	Slo2 channel	Slo2 channel	Last 15 years	3

Results

Database and Search Strategy

**Figure 1:**
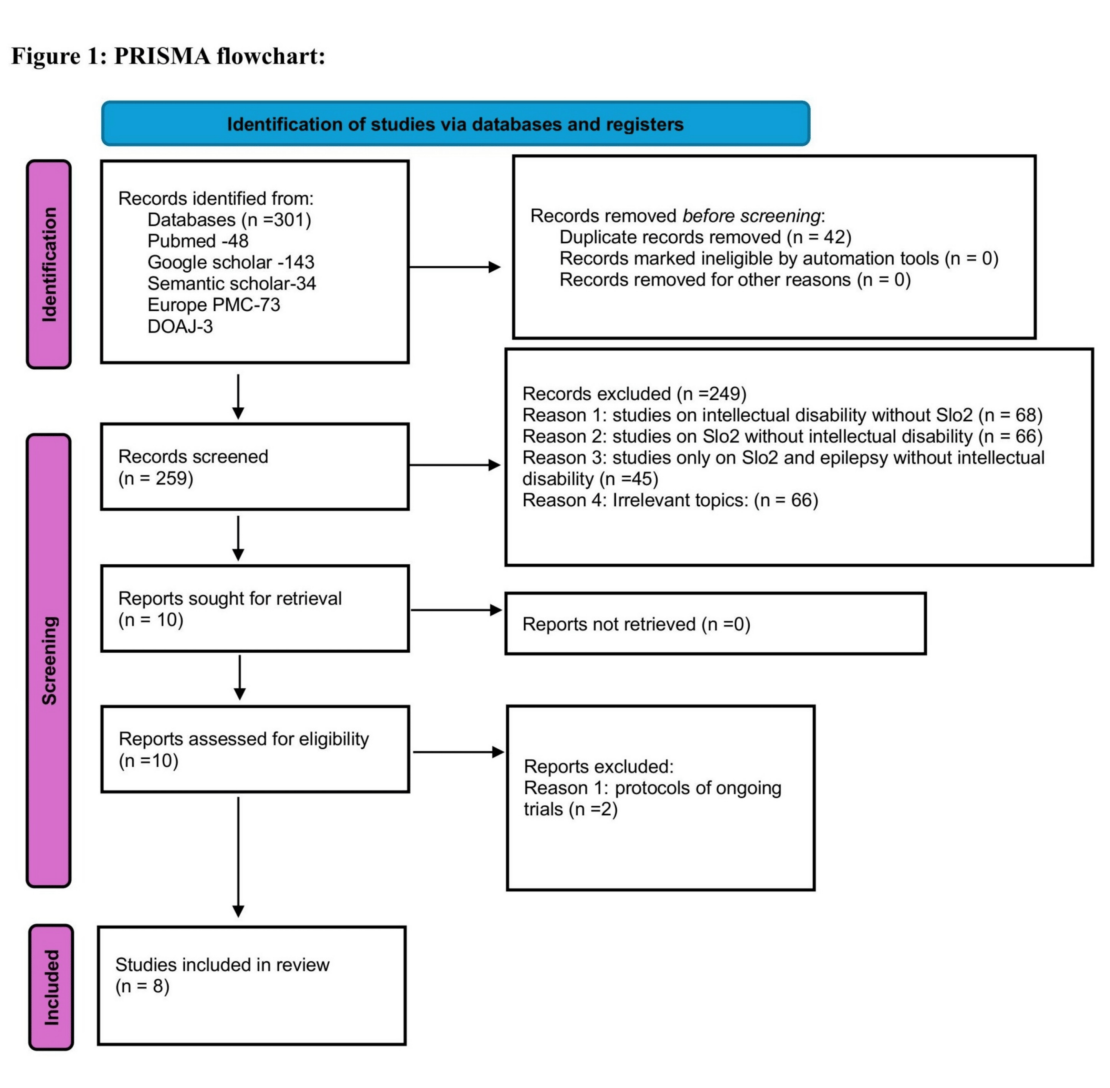
PRISMA flowchart showing the complete selection and screening process PMC: PubMed Central, DOAJ: Directory of Open Access Journals.

A comprehensive literature search across databases including PubMed, Europe PubMed Central, Semantic Scholar, DOAJ, and Google Scholar yielded 301 articles, of which 42 duplicates were removed. The remaining 259 articles underwent title and abstract screening, resulting in the exclusion of 249 studies. These studies were categorized as follows: 68 did not include Slo2 and were on ID, 66 did not include Slo2 and were on ID, 45 included Slo2 and epilepsy but not ID, and 66 were deemed irrelevant. Ten articles were retrieved for full-text assessment, and two were excluded as they were protocols for ongoing trials. The remaining eight articles were evaluated for eligibility and quality using appropriate appraisal tools. Only studies achieving a quality score of 70% or more were included in the systematic review, resulting in the inclusion of all eight articles. The complete selection and screening process, along with excluded studies and reasons for exclusion, is summarized in the PRISMA flowchart (Figure 1).

Quality Assessment Tools

Studies were preliminarily assessed by examining titles and abstracts to filter out ineligible articles. Subsequently, full-text articles of the selected records were retrieved for a more detailed evaluation. The remaining full-text articles were evaluated for quality and risk of bias using study-specific tools. AMSTAR 2 was applied for systematic reviews, cell study-OHAT-inspired for cell experimental studies, and SYRCLE RoB for animal studies. Each assessment tool had its own criteria and scoring system. A minimum score of 70% on each tool was considered acceptable. Description of the quality assessment of studies is given in Table 2.

**Table 2:** Quality assessment tools This table presents details of experimental and review studies investigating Slo2 channels and KCNT1/KCNT2-related neurodevelopmental disorders. It includes the type of model or study design, the risk of bias or quality assessment tool used (e.g., SYRCLE RoB, AMSTAR 2, OHAT-inspired), overall confidence rating, and important observations or limitations for each study.

Study	Model type	RoB/quality tool	Overall confidence	Key notes
Zhang et al. (2012) [6]	Aplysia neurons, xenopus oocytes	Cell study-OHAT-inspired	Moderate	Single experimental study, in vitro; no behavioral data; some detection bias possible
Kim and Kaczmarek (2014) [9]	Systematic review	AMSTAR 2	Critically low	Narrative synthesis; critical domains not met; lacks experimental validation; focus on epilepsy
Bausch et al. (2015) [11]	Kcnt1 null mice	SYRCLE RoB	Moderate	Well-designed behavioral study; unclear allocation concealment; partial blinding of outcome assessment
Quraishi et al. (2020) [12]	Kcnt1 -/- and R455H mice	SYRCLE RoB	Low	Adult animal models, non-standard seizure protocols; genetic background differences; blinding not reported
Kessi et al. (2020) [13]	Systematic review	AMSTAR 2	Low	Comprehensive literature search, but critical domains partially met; lack of experimental validation; heterogeneous data
Gong et al. (2021) [14]	Case report (KCNT2 variants)	N/A (case report)	Very low	Only two patients; small sample size; genotype-phenotype correlations uncertain; observational design
Wu et al. (2024) [15]	Rat PFC neurons	SYRCLE RoB	Moderate	Electrophysiology + behavioral testing; small sample; partial blinding; direct functional outcomes assessed
Malone et al. (2025 [16]	HEK cells, primary cortical neurons	Cell study-OHAT-inspired	Moderate	Mechanistic insights; potential off-target effects; detection bias in translation assays

Meta-analysis and Quantitative Synthesis

Meta-analysis and quantitative synthesis were not performed due to substantial heterogeneity across the included studies.

Analysis

Study characteristics: Eight articles that met the quality assessment criteria, comprising five experimental studies, two systematic reviews, and one case report, were included in this study design (Table 3).

**Table 3:** Study characteristics This table summarizes the included studies: study type, model systems, methodologies, key results, and limitations and provides an overview of experimental studies, systematic reviews, and case reports investigating the role of Slo2/KCNT1/KCNT2 channels in intellectual disability and related neurodevelopmental disorders. It details the type of study, model system or population used, primary methodologies employed, main findings, and notable limitations for each study, highlighting strengths, potential biases, and gaps in the existing literature.

Study	Study type	Model system	Methodology	Results	Limitation
Zhang et al. (2012) [6]	Experimental study	Aplysia bag cell neurons, xenopus oocytes	Immunocytochemistry and coimmunoprecipitation to demonstrate FMRP and Slack interaction. RNA interference (siRNA) to suppress Slack expression. Cloning of Aplysia Slack using PCR from a cDNA library. Electrophysiological techniques. Intracellular injection of FMRP to observe effects on outward currents. Biphasic outward current measured	FMRP and Slack channels interact, and FMRP enhances Slack channel activity. The interaction may link neuronal firing to protein translation, influencing long-term changes in neuronal excitability. No direct behavioral assessment	The current findings do not eliminate alternative hypotheses. Further research is needed to identify proteins involved in excitability changes
Kim and Kaczmarek (2014) [9]	Systematic review	Systematic review	The paper is a review article on Slack channels and their role in intellectual disability related to epilepsy, including malignant migrating partial seizures in infancy, autosomal dominant nocturnal frontal lobe epilepsy, and Ohtahara syndrome, compiling data from various studies	Slack channels are associated with intellectual disability in fragile X syndrome and are linked to severe delays in cognitive development in early-onset epileptic encephalopathies	Limited understanding of how Slack channel activity is altered in different conditions. Hypotheses about mechanisms are not experimentally tested
Bausch et al. (2015) [11]	Experimental study	Kcnt1 null mice and wild type	Use of the Kcnt1 null mouse model to assess Slack channel role in memory and learning. Morris water maze (MWM) task for spatial learning and reversal learning. Open-field test for explorative motivation and locomotor activity. Anxiety behavior by the dark light box test. Circular maze for reference and reversal learning	The study demonstrates that Slack channels are required for cognitive flexibility, spatial learning, and adapting to new situations, are involved in regulating anxiety-like behaviors in mice, but not for working memory or reference memory. Similar results have been seen in FMRP-deficient mice	Cellular mechanisms affected by Slack deficiency in reversal learning and cognitive flexibility are not well understood. Impact of Slack deficiency on bidirectional synaptic plasticity needs further investigation
Quraishi et al. (2020) [12]	Experimental study	Kcnt1 -/- mice, heterozygous Kcnt1 +/R455H mice, and wild-type mice	Creation of mouse models with deletion of the Kcnt1 gene and the R455H mutation using CRISPR/Cas9. EEG electrode implantation for seizure monitoring. Electroshock and PTZ chemical induction for seizure susceptibility testing. Behavioral tests: open-field activity, rotarod for motor skill learning, and Lashley III maze for spatial learning. Video EEG monitoring for spontaneous seizures	Complete loss of Kcnt1 produced deficits in open-field behavior, motor skill learning, and anxiety-like behavior, but no defect in spatial learning. Kcnt1 -/- mice were protected from death after maximum electroshock-induced seizures. Heterozygous Kcnt1 +/R455H mice had increased seizure susceptibility and activity, supporting the hypothesis that increased K Na current leads to epilepsy	The study tested adult animals, but the human disease presents in infancy. The knockout and knock-in mice were on different background strains and were compared to their separate wild types, but not compared directly to each other. The seizure induction tests did not follow the NINDS antiepileptic drug screening program protocol
Kessi et al. (2020) [13]	Systematic review	Systematic review	Conducted a systematic review following PRISMA guidelines. Searched PubMed and Embase databases up to October 2019. Used specific search strategies focusing on intellectual disability and potassium channelopathies. Included cohorts, case-controls, cross-sectionals, case series, and case reports. Selected studies with patients having ID/GDD and potassium channel gene mutations. Excluded studies with other channelopathies or gene mutations	Potassium channelopathies play a significant role in initiating intellectual disability (ID), with both gain-and loss-of-function mutations leading to ID. A total of 19 potassium channelopathies affecting various genes are associated with ID. The mechanisms by which gain-and loss-of-function mutations lead to ID are not fully understood, necessitating further research	Lack of available information on distinct animal models. Paucity of animal studies on the mechanisms of ID related to potassium channelopathies. Limited benefits of available treatment options (channel openers or blockers). Need for further research on the effects of gain-and loss-of-function mutations. Need for additional research on interactions between channels and other proteins. Limited understanding of gene therapies/editing as potential treatments
Gong et al. (2021) [14]	Case report	Case report	Whole-exome sequencing to identify likely pathogenic variants in KCNT2 causing developmental and epileptic encephalopathies (DEEs). Retrospective collection and analysis of clinical data. Confirmation of variants using conventional Sanger sequencing. Pathogenicity analysis using PolyPhen-2 and MutationTaster. Classification of variants as likely pathogenic based on the American College of Medical Genetics (ACMG) guidelines	The study presents detailed clinical features and genetic analysis of two patients with KCNT2-related DEE, expanding the known spectrum of KCNT2 mutations. IS and EIMFS are identified as the most common phenotypes caused by pathogenic mutations in KCNT2. Both gain- and loss-of-function mutations can lead to EIMFS, and quinidine is suggested as a potential personalized medicine approach for KCNT2-related DEE	Small sample size, difficulty in making genotype-phenotype correlations, need for additional cases to clarify correlations, need for further clinical follow-up to understand long-term effects of quinidine
Wu et al. (2024) [15]	Experimental study	Rat PFC neurons	HCN and Slack channels co-immunoprecipitate and colocalize at postsynaptic spines of PFC pyramidal neurons. Whole-cell voltage clamp recordings for electrophysiological assessment. Behavioral experiments using the delayed alternation test for spatial working memory. Stereotaxic surgery for drug infusion	The regulation of working memory by HCN channels in PFC pyramidal neurons is mediated by an HCN-Slack channel complex that links activation of HCN channels to suppression of neuronal excitability. Blocking either HCN or Slack channels improves working memory performance in rats. The regulation of working memory by HCN channels is mediated by an HCN-Slack channel complex that suppresses neuronal excitability	Further genetic studies are needed to establish the specific function of the HCN-Slack channel complex in working memory regulation
Malone et al. (2025 [16]	Experimental study	Cell lines and primary cortical neurons	Constitutively active Slack mutation and pharmacological stimulation. Immunoprecipitation to study Slack-FMRP/CYFIP1 interaction. Reporter construct (dendra2-actin) for translation measurement. Puromycin incorporation assays for translation regulation. FRAP for real-time dendra2-actin fluorescence monitoring. Experiments in HEK cells and primary cortical neurons using siRNA for FMRP and CYFIP1 knockdown to study translation	Activation of Slack potassium channels triggers the translocation of FMRP and CYFIP1 from eIF4E, stimulating mRNA translation initiation in both cell lines and neurons, affecting the synthesis of β-actin. Dysregulation of translation by Slack mutations may contribute to intellectual disability	Potential off-target effects of quinidine cannot be concluded on K flux and translation. Discrepancy in measuring fluorescence versus protein levels

Table 4 summarizes the key findings of studies on Slo2/KCNT1/KCNT2 channels in ID, with GRADE ratings and rationale. 

**Table 4:** GRADE assessment of evidence linking Slo2 potassium channels to cognitive impairment in intellectual disability This table summarizes the key findings of studies on Slo2/KCNT1/KCNT2 channels in intellectual disability, with GRADE ratings and rationale highlighting limitations, sample size, model relevance, and translational applicability.

Study	Outcome/findings	GRADE certainty	Rationale
Zhang et al. (2012) [6]	FMRP enhances Slack channel activity	Low	Single study, in vitro/animal data only, no behavioral assessment, indirect relevance to human cognition
Kim and Kaczmarek (2014) [9]	Slack channels linked to ID in epilepsy	Low-moderate	Review compiles evidence; mechanisms not experimentally tested, indirect focus on epilepsy rather than cognition
Bausch et al. (2015) [11]	Slack channels required for cognitive flexibility and anxiety regulation	Moderate	Animal behavioral data supports cognition role; mechanisms underlying bidirectional plasticity unclear; limited translation to humans
Quraishi et al. (2020) [12]	Kcnt1 deletion/mutation affects behavior, seizure susceptibility	Low-moderate	Adult animal models, different genetic backgrounds, seizure protocols non-standard, indirect relevance to infancy-onset human disease
Kessi et al. (2020) [13]	Potassium channelopathies contribute to ID; gain/loss mutations implicated	Low	Mechanistic understanding limited; lack of animal models; sparse therapeutic data; mostly descriptive
Gong et al. (2021) [14]	KCNT2 de novo variants cause DEE; quinidine potential therapy	Very low	Only two patients; small sample; genotype-phenotype correlation limited; long-term effects unknown
Wu et al. (2024) [15]	HCN-Slack complex regulates PFC excitability and working memory	Low-moderate	Mechanistic and behavioral evidence present, but preclinical, small sample, limited generalizability
Malone et al. (2025 [16]	Slack mutations affect FMRP-mediated translation, may contribute to ID	Low-moderate	Mechanistic insights strong, but in vitro models; off-target effects and measurement discrepancies reduce certainty

Table 5 summarizes the primary and secondary outcomes. 

**Table 5:** Summary of primary and secondary outcomes with planned synthesis approach

Outcome (primary/secondary)	Model/study	Direction of effect	Mechanistic insight	Evidence type/certainty
Cognitive flexibility/reversal learning	Kcnt1 null mice [11]	Impaired	Slack channels required for adapting to new situations; regulates anxiety-like behavior	Experimental, animal/moderate
Spatial learning/reference memory	Kcnt1 -/- mice [12]	No defect	Kcnt1 deletion protects from seizures; adult models may not represent infancy-onset disease	Experimental, animal/low-moderate
Working memory	Rat PFC neurons [15]	Improved with HCN-Slack blockade	HCN-Slack complex suppresses neuronal excitability; links channel function to working memory	Experimental, animal/moderate
FMRP translation/protein synthesis	HEK cells, primary neurons [16]	Increased translation with Slack activation	Slack-FMRP/CYFIP1 dissociation promotes mRNA translation; affects β-actin synthesis	Experimental, cell/moderate
FMRP and Slack interaction	Aplysia neurons, xenopus oocytes [6]	FMRP enhances Slack activity	Interaction may link neuronal firing to protein translation, influencing excitability	Experimental, cell/low
ID linked to epilepsy/Slack channels	Systematic review [9]	Association reported	Slack channels linked to cognitive delays in early-onset epileptic encephalopathies	Systematic review, AMSTAR 2/critically low
Potassium channelopathies and ID	Systematic review [13]	Gain-/loss-of-function mutations lead to ID	Mechanisms not fully understood; 19 channelopathies identified	Systematic review, AMSTAR 2/low
Human genotype-phenotype (KCNT2 variants)	Case report [14]	ID + seizures	Gain/loss mutations affect excitability; quinidine as potential therapy	Case report/very low

Discussion

Sequences like the calcium-activated potassium channel (Slack channel), also known as KCNT1 or Slo2.2, and KCNT2 (Slick/Slo2.1) are sodium-activated potassium channels. KCNT mRNA and proteins are expressed in high numbers in neurons and regulate neuronal excitability [9,17-19]. Researchers over the past decade have found a mutation in the Slack channel associated with severe early-onset epilepsy and ID [11]. Mutations in the Slick channel have been linked to ID associated with several severe epilepsy syndromes, including early-onset epileptic encephalopathy, West syndrome progressing to Lennox-Gastaut syndrome, and epilepsy of infancy with migrating focal seizures [12,18,20]. Given the wide-reaching effects of Slack and Slick channel mutations, understanding their molecular mechanisms is crucial for linking channelopathies to clinical outcomes. In particular, the interplay between these channels and regulatory proteins can provide insight into how neuronal development is shaped at the molecular level. This perspective helps reveal how disruptions in these pathways may translate into specific neurodevelopmental disorders.

Fragile X syndrome is the most common cause of ID in humans, which occurs due to loss of FMRP. The interaction between Slack potassium channels and FMRP is evolutionarily conserved. It may link changes in neuronal firing to changes in protein translation, playing a role in the development of developmental delays. Investigating the interaction between FMRP and Slack in Aplysia Bag cell neurons using immunocytochemistry and coimmunoprecipitation showed that FMRP and Slack channels colocalize, indicating the possibility of a functional relationship. Injecting FMRP lacking its mRNA-binding domain induced a biphasic outward current in the Slack channels. FMRP also increased Slack channel opening, raising the possibility that FMRP interaction with Slack may alter protein synthesis and neuronal development [6]. Understanding these molecular interactions provides a foundation for examining how mutations in Slack channels impact cognitive development.

Building upon this foundation, subsequent investigations identified that Slack channels are implicated in ID; notably, mutations in the KCNT1 gene, which encodes Slack, have been found in early-onset epileptic encephalopathies, where they are associated with severe cognitive delays. The SLACK channel has been implicated in ID-related epilepsies, including malignant migrating partial seizures of infancy, autosomal dominant nocturnal frontal lobe epilepsy, and Ohtahara syndrome [9,13,14]. Slack mutation and FMRP regulation of Sack channel cause a significant reduction of sub-conductance state, producing rapid repolarization and shortening of action potential duration, indirectly increasing cell excitability [9]. This connection supported the hypothesis that dysregulated Slack activity may contribute not only to epilepsy but also to developmental and intellectual impairments. To build a more complete picture, it is important to examine behavioral studies that reveal the functional consequences of Slack channel mutations.

Further behavioral studies in animal models revealed that Slack channels are critical for cognitive flexibility, spatial learning, and behavioral adaptation to new environments. In Fragile X syndrome, reduced K⁺ currents in hippocampal pyramidal neurons alter dendritic signaling, impairing synaptic function and causing learning and memory deficits [21]. Morris water maze (MWM) and the open-field test for learning and locomotor activity showed that Slack channel mutation causes changes in locomotor activity, but does not affect working memory, reference memory, and motor coordination. Slack channels are found to be essential for cognitive flexibility, reversal learning, adaptation to new environments, and hippocampal-dependent spatial learning. Slack channels deficiency leads to altered anxiety-like behaviors and learning deficits, reinforcing the functional linkage between these two proteins [22,23]. Insights gained from these models help clarify how molecular changes can impact behavior and cognition in neurodevelopmental disorders. Appreciating these behavioral changes is key to understanding the full scope of disorders linked to Slack dysfunction.

Both loss-of-function and gain-of-function mutations are associated with ID [24]. The study demonstrated the association between Slack channel mutation, procedural learning, and seizure susceptibility. In models with complete Kcnt1 knockout, mice exhibited abnormal motor skill learning, altered open-field behavior, and anxiety-like traits, although spatial learning remained unaffected. Remarkably, these knockout mice were resistant to seizure-induced death. In contrast, mice with a gain-of-function Kcnt1 mutation (R455H) showed increased seizure susceptibility and heightened neuronal activity, underscoring the delicate balance of potassium channel activity in regulating excitability and seizure thresholds [25]. A deeper investigation into protein interactions helps explain how Slack channel mutations produce such diverse effects.

Slack channels are known to interact with key regulatory proteins, including FMRP, Phactr1, and CYFIP1. The interaction between FMRP and Slack influences the likelihood of channel opening, while mutations in Slack channels disrupt their interaction with Phactr1 entirely. This loss of interaction may contribute to the development of severe ID and epilepsy. Therefore, FMRP, Phactr1, and CYFIP1 represent promising targets for novel therapeutic approaches, while future studies using animal models may help uncover the roles of additional proteins involved in the pathology [5,6,8]. Investigating these molecular partnerships can expand our understanding of the pathogenesis of ID.

Potassium channelopathies are linked to ID, with both gain- and loss-of-function mutations implicated, although the underlying mechanisms remain unclear [26]. Activation of Slack potassium channels triggers the translocation of FMRP and CYFIP1 from eIF4E, stimulating mRNA translation [27], and mutations in Slack may contribute to ID by dysregulating this process. Gain-of-function mutations in Slack channels can lead to autosomal dominant nocturnal frontal lobe epilepsy (ADNFLE), a condition strongly associated with developmental delays and a high risk of ID in affected individuals. Increased binding of the FMRP/CYFIP1 complex to Slack channels enhances translation initiation of β-actin, both in neurons and in cell lines expressing Slack, which is dependent on the UTR regions of the β-actin gene and is blocked when FMRP or CYFIP1 expression is reduced. Since β-actin is crucial for synaptic plasticity and is translated in response to synaptic activity, the findings suggest that impaired activity-dependent protein synthesis, rather than seizures alone, plays a key role in the ID observed in Slack channel mutations. This supports the broader view that translational dysregulation is a critical contributor to cognitive impairment [27,28].

Spatial working memory in the prefrontal cortex (PFC) depends on persistent firing of pyramidal neurons that express hyperpolarization-activated cyclic nucleotide-gated (HCN) channels, which are regulated by cyclic adenosine monophosphate (cAMP). Activation of HCN channels by cAMP reduces working memory-related neuronal firing by stimulating sodium-activated potassium (Slack) channels. This process forms a postsynaptic HCN-Slack complex that suppresses neuronal excitability. Pharmacological inhibition of either HCN or Slack channels improves working memory in rats, suggesting a shared regulatory pathway and underscoring the HCN-Slack complex as a critical mediator of PFC-dependent cognitive function [15]. Insights into these signaling pathways further illuminate the complexities of neuronal network regulation.

Slack channels are essential for regulating the timing of high-frequency neuronal firing. Loss of Slack channel function in medial nucleus of the trapezoid body (MNTB) neurons may impair signal processing and contribute to cognitive and auditory deficits [29]. Screening of 362 patients with early-onset epileptic encephalopathies showed nine heterozygous KCNT1 mutations, predominantly de novo, clustered in the regulator of conductance for potassium (RCK) domain and transmembrane segment 5, with several recurrent variants. KCNT1 mutations were present in approximately 50% of epilepsy of infancy with migrating focal seizures (EIMFS) cases, indicating that KCNT1 is a primary genetic cause of this disorder [30]. Findings like these emphasize the genetic landscape underlying severe epilepsy syndromes.

Two de novo heterozygous KCNT1 mutations were identified in unrelated malignant migrating partial seizures of infancy (MMPSI) patients. Functional studies demonstrated enhanced channel currents and leftward shifts in activation gating, consistent with gain-of-function effects [31]. Exome sequencing of 12 unrelated MMPSI patients in another study revealed de novo gain-of-function mutations in the C-terminal domain of KCNT1 in six individuals. Functional assays showed constitutive channel activation, which mimics protein kinase C phosphorylation and may disrupt developmental signaling through C-terminal interactions. These findings highlight KCNT1 mutations as a key molecular mechanism in MMPSI and provide potential targets for diagnosis and therapy [32].

Alterations in KCNT1 or Slack channels disrupt the excitatory-inhibitory balance, contributing to cognitive deficits and neurological or psychiatric disorders. This underscores the urgent need for novel therapeutic strategies targeting these channels [33]. Notably, the KCNT1 blockers quinidine and bepridil inhibited mutant channels more effectively than wild-type channels, suggesting potential genotype-specific therapeutic approaches [31,34]. The spectrum of disorders associated with potassium channel dysfunction is rapidly expanding, particularly in neuroscience. The diverse roles and widespread distribution of these channels offer promising therapeutic opportunities, but also present challenges due to potential off-target effects [34]. A comprehensive understanding of potassium channelopathies is crucial for directing future research and patient care. Continued interdisciplinary collaboration will be key to translating scientific discoveries into effective treatments.

Limitations

The mechanisms underlying alterations in Slack channel activity remain undefined, and most proposed explanations lack experimental validation. Most studies utilized adult animal models, although the human disease manifests in infancy. Pharmacological interventions, including channel openers and blockers, have demonstrated limited efficacy. Gene therapy and gene editing approaches are still in the early stages of development. The effects of quinidine on potassium ion flux and protein translation remain incompletely characterized. Small sample sizes, inconsistent measurement methods, and limited clinical follow-up hinder the establishment of robust genotype-phenotype correlations and the assessment of long-term treatment outcomes. Addressing these limitations will require larger, standardized, and longitudinal studies.

## Conclusions

This systematic review synthesizes recent evidence to provide an up-to-date overview of Slo2 channel research. Only studies that met established quality appraisal criteria were included, which strengthens the validity of the findings. Slo2 channels were analyzed in relation to both gain- and loss-of-function mutations. The evidence synthesized in this review underscores the importance of further studies and advancing therapeutic strategies that directly target Slo2 channels, particularly in the context of gain-of-function mutations. While existing data provide preliminary support for Slo2 inhibition as a promising approach, substantial gaps remain in both the pharmacological development of inhibitors and their clinical validation.

A critical next step will be the design of inhibitors with high target specificity and adequate penetration into the central nervous system, ensuring that compounds effectively reach and modulate their intended targets without producing significant off-target effects. In addition, evaluating mutation-specific effects will be essential for translating Slack-targeted therapies into precision medicine approaches, as different KCNT1 variants may produce variable functional consequences and treatment responses. Such studies will be crucial in moving beyond proof-of-concept evidence and toward the development of viable therapeutic options for devastating conditions that remain refractory to conventional anti-seizure medications. Slack channels represent a novel and compelling target in epilepsy drug discovery, but translating this potential into clinical practice will require a coordinated effort that spans drug design, precision medicine, and clinical testing. Progress in these areas could ultimately open new avenues for treating KCNT1-related epilepsies and other neurological disorders linked to neuronal hyperexcitability.

## References

[1] Damtie Y, Dachew BA, Ayano G, Tadesse AW, Betts K, Alati R (2025). The risk of intellectual disability in offspring of diabetic mothers: a systematic review and meta-analysis. J Psychosom Res.

[2] Santoro MR, Bray SM, Warren ST (2012). Molecular mechanisms of fragile X syndrome: a twenty-year perspective. Annu Rev Pathol.

[3] Devys D, Lutz Y, Rouyer N, Bellocq JP, Mandel JL (1993). The FMR-1 protein is cytoplasmic, most abundant in neurons and appears normal in carriers of a fragile X premutation. Nat Genet.

[4] Bassell GJ, Warren ST (2008). Fragile X syndrome: loss of local mRNA regulation alters synaptic development and function. Neuron.

[5] Brown MR, Kronengold J, Gazula VR (2010). Fragile X mental retardation protein controls gating of the sodium-activated potassium channel Slack. Nat Neurosci.

[6] Zhang Y, Brown MR, Hyland C (2012). Regulation of neuronal excitability by interaction of fragile X mental retardation protein with slack potassium channels. J Neurosci.

[7] Tamsett TJ, Picchione KE, Bhattacharjee A (2009). NAD+ activates KNa channels in dorsal root ganglion neurons. J Neurosci.

[8] Fleming MR, Brown MR, Kronengold J (2016). Stimulation of Slack K+ channels alters mass at the plasma membrane by triggering dissociation of a phosphatase-regulatory complex. Cell Rep.

[9] Kim GE, Kaczmarek LK (2014). Emerging role of the KCNT1 Slack channel in intellectual disability. Front Cell Neurosci.

[10] Zhang J, Liu S, Fan J (2023). Structural basis of human Slo2.2 channel gating and modulation. Cell Rep.

[11] Bausch AE, Dieter R, Nann Y (2015). The sodium-activated potassium channel Slack is required for optimal cognitive flexibility in mice. Learn Mem.

[12] Quraishi IH, Mercier MR, McClure H (2020). Impaired motor skill learning and altered seizure susceptibility in mice with loss or gain of function of the Kcnt1 gene encoding Slack (K(Na)1.1) Na(+)-activated K(+) channels. Sci Rep.

[13] Kessi M, Chen B, Peng J (2020). Intellectual disability and potassium channelopathies: a systematic review. Front Genet.

[14] Gong P, Jiao X, Yu D, Yang Z (2021). Case report: Causative de novo variants of KCNT2 for developmental and epileptic encephalopathy. Front Genet.

[15] Wu J, El-Hassar L, Datta D (2024). Interaction between HCN and slack channels regulates mPFC pyramidal cell excitability in working memory circuits. Mol Neurobiol.

[16] Malone TJ, Wu J, Zhang Y (2025). Neuronal potassium channel activity triggers initiation of mRNA translation through binding of translation regulators. Sci Adv.

[17] Bhattacharjee A, Gan L, Kaczmarek LK (2002). Localization of the Slack potassium channel in the rat central nervous system. J Comp Neurol.

[18] Ambrosino P, Soldovieri MV, Bast T (2018). De novo gain-of-function variants in KCNT2 as a novel cause of developmental and epileptic encephalopathy. Ann Neurol.

[19] Mao X, Bruneau N, Gao Q (2020). The epilepsy of infancy with migrating focal seizures: identification of de novo mutations of the KCNT2 gene that exert inhibitory effects on the corresponding heteromeric K(Na)1.1/K(Na)1.2 potassium channel. Front Cell Neurosci.

[20] Martinez-Espinosa PL, Wu J, Yang C (2015). Knockout of Slo2.2 enhances itch, abolishes KNa current, and increases action potential firing frequency in DRG neurons. Elife.

[21] Bausch AE, Ehinger R, Straubinger J, Zerfass P, Nann Y, Lukowski R (2018). Loss of sodium-activated potassium channel slack and FMRP differentially affect social behavior in mice. Neuroscience.

[22] Ishii A, Shioda M, Okumura A (2013). A recurrent KCNT1 mutation in two sporadic cases with malignant migrating partial seizures in infancy. Gene.

[23] Routh BN, Johnston D, Brager DH (2013). Loss of functional A-type potassium channels in the dendrites of CA1 pyramidal neurons from a mouse model of fragile X syndrome. J Neurosci.

[24] Ohba C, Kato M, Takahashi N (2015). De novo KCNT1 mutations in early-onset epileptic encephalopathy. Epilepsia.

[25] Brager DH, Johnston D (2014). Channelopathies and dendritic dysfunction in fragile X syndrome. Brain Res Bull.

[26] Rizzo F, Ambrosino P, Guacci A (2016). Characterization of two de novoKCNT1 mutations in children with malignant migrating partial seizures in infancy. Mol Cell Neurosci.

[27] Barcia G, Fleming MR, Deligniere A (2012). De novo gain-of-function KCNT1 channel mutations cause malignant migrating partial seizures of infancy. Nat Genet.

[28] Faulkner IE, Pajak RZ, Harte MK, Glazier JD, Hager R (2024). Voltage-gated potassium channels as a potential therapeutic target for the treatment of neurological and psychiatric disorders. Front Cell Neurosci.

[29] Alam KA, Svalastoga P, Martinez A, Glennon JC, Haavik J (2023). Potassium channels in behavioral brain disorders. Molecular mechanisms and therapeutic potential: a narrative review. Neurosci Biobehav Rev.

[30] Kaczmarek LK (2013). Slack, slick and sodium-activated potassium channels. ISRN Neurosci.

[31] Tang QY, Zhang FF, Xu J, Wang R, Chen J, Logothetis DE, Zhang Z (2016). Epilepsy-related slack channel mutants lead to channel over-activity by two different mechanisms. Cell Rep.

[32] Møller RS, Heron SE, Larsen LH (2015). Mutations in KCNT1 cause a spectrum of focal epilepsies. Epilepsia.

[33] Rizzi S, Schwarzer C, Kremser L, Lindner HH, Knaus HG (2015). Identification of potential novel interaction partners of the sodium-activated potassium channels Slick and Slack in mouse brain. Biochem Biophys Rep.

[34] Heron SE, Smith KR, Bahlo M (2012). Missense mutations in the sodium-gated potassium channel gene KCNT1 cause severe autosomal dominant nocturnal frontal lobe epilepsy. Nat Genet.

